# Facilitators and barriers to using telepresence robots in aged care settings: A scoping review

**DOI:** 10.1177/20556683211072385

**Published:** 2022-01-21

**Authors:** Lillian Hung, Joey Wong, Chelsea Smith, Annette Berndt, Mario Gregorio, Neil Horne, Lynn Jackson, Jim Mann, Mineko Wada, Erika Young

**Affiliations:** 1 School of Nursing, 8166 University of British Columbia, Vancouver, BC, Canada; 2 Community Engagement Advisory Network, Vancouver, BC, Canada

**Keywords:** Dementia, robots, telepresence, technology, implementation

## Abstract

Social isolation has been a significant issue in aged care settings, particularly during the COVID-19 pandemic, and is associated with adverse outcomes, including loneliness, depression, and cognitive decline. While robotic assistance may help mitigate social isolation, it would be helpful to know how to adopt technology in aged care. This scoping review aims to explore facilitators and barriers to the implementation of telepresence robots in aged care settings. Following the Joanna Briggs Institute scoping review methodology and the PRISMA extension for scoping reviews reporting guidelines, we searched relevant peer-reviewed studies through eight databases: CINAHL, MEDLINE, Cochrane, PsychINFO (EBSCO), Web of Science, ProQuest Dissertations and Theses Global, IEEE Xplore, and ACM Digital Library. Google was used to search gray literature, including descriptive, evaluative, quantitative, and qualitative designs. Eligibility includes: studies with people aged 65 years and older who interacted with a telepresence robot in a care setting, and articles written in English. We conducted a thematic analysis to summarize the evidence based on the constructs in the Consolidated Framework of Implementation Research. Of 1183 articles retrieved, 13 were included in the final review. The analysis yielded three themes: relative advantages, perceived risks and problems, and contextual considerations. The key facilitators to telepresence robot adoption are as follows: a feeling of physical presence, ease of use, mobility, and training. The barriers to implementation are as follows: cost, privacy issues, internet connectivity, and workflow. Future research should investigate the role of leadership support in implementation and practical strategies to overcome barriers to technology adoption in aged care settings.

## Introduction

### Social isolation in aged care setting

As older adults in aged care settings (e.g., hospitals and long-term care) in Canada have been disproportionately impacted by the COVID-19 outbreaks, they are more immensely at risk of social isolation than before, as shown in Canada’s national seniors strategy report.^1^ Social isolation commonly refers to low quantity and quality of contact. Individuals who are socially isolated have few social contacts, social roles, and an absence of mutually rewarding social relationships.^2^ Research indicates that social isolation is associated with adverse outcomes such as loneliness, depression, cognitive decline, and mortality.^1^ The physical distancing and visitation restrictions during the COVID-19 pandemic have exacerbated social isolation among older adults in the aged care settings.^1^ Although technologies like smartphones and virtual online communication platforms have become more commonly used to facilitate social connections, it is difficult for older adults in aged care settings to connect with their family and friends virtually using these technologies due to lack of skills, multiple morbidities, and functional limitations. The social exclusion created by technologies has worsened the impact of the pandemic on older adults in aged care settings during this challenging time.^3^ Technology that is adapted for older adults is needed to alleviate this problem.

### Telepresence robots for social connections in aged care settings

**Figure 1.**
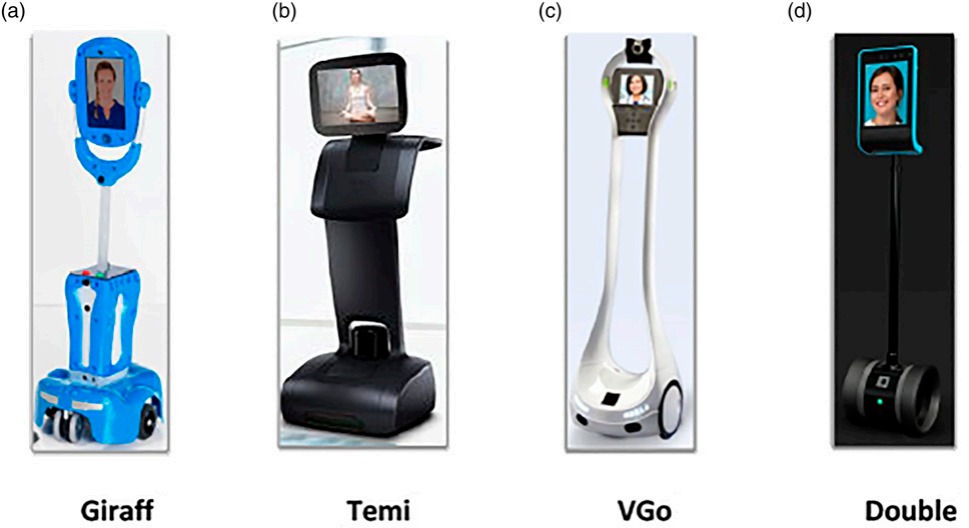
Examples of telepresence robots. (a) Giraff,^6^ (b)Temi,^7^ (c) VGo,^8^ and (d) Double.^9^.

Telepresence robots allow videoconferencing for real-time communication and consist of wheels for movement (Figure 1).^4^ These robots have been used for various situations such as remote learning^5^ and office meetings;^4^ however, there is a growth of robotic use in aged care settings to support social connections and increase the quality of life of older adults. Given the visitor restrictions and social isolation in aged care settings, telepresence robots have the potential to mitigate the impact of isolation by supporting safe social connections. The design of telepresence robots facilitates remote face-to-face interactions between family members and older persons and enables the robots to move around the care environment. Telepresence robots can be controlled remotely (e.g., by distant family members) via wireless connection to the internet. The remote-controlled function alleviates technical challenges for older adults as they do not need to learn and operate technologies by themselves. Overall, telepresence robots allow individuals to have social connections with remote family members.

### Acceptance of telepresence robots

As new technology and innovation, the acceptance of telepresence robots by users and stakeholders is crucial to technology adoption. Previous research investigating the acceptance of telepresence robots of potential users (older adults, family members, and health professionals) indicated that telepresence robots are generally accepted by these stakeholders.^10–12^ Some research identified factors that contribute to user acceptance of telepresence robots, including usability and potential to increase social contact.^12,13^ For example, if older adults perceive themselves as competent to handle the robots, their acceptance of the robots is associated with their view that the robots fulfill their social needs.^13^ If older adults view themselves as unable to use the robots independently, their acceptance is associated with their perceived social and psychological resources to handle the robots.^13^ While older adults showed positive acceptance of telepresence robots and interests to have them for an extended period after research,^11^ future research is warranted to investigate long-term acceptance.^12^

### Implementation of telepresence robots in aged care settings

Despite the potentials, benefits and acceptance of telepresence robots in aged care settings, the strategies for their successful implementation in aged care settings (e.g., long-term care homes) remain unclear. A review published in 2017 focused on the use of telepresence robots to enhance social connectedness in older adults with dementia.^14^ The review highlighted positive outcomes of using telepresence robots to connect people with dementia to others via videoconferencing, which helped guide the development of this scoping review. We aimed to build upon the 2017 review by including more recent publications focusing on implementation strategies with the guidance of a systematic framework. The objective of this review was to synthesize and discuss evidence to address the research question: what has been identified as facilitators and barriers to using telepresence robots among older people in care settings? This review also offered recommendations and implications for implementing telepresence robots in aged care settings.

## Methods

Scoping reviews are useful to systematically map and synthesize the current state of evidence when a research topic is new and has not been fully established.^15^ This scoping review followed the key guidance and reporting standards in the field, including the Joanna Briggs Institute (JBI) methodological guidance for scoping reviews^15^ and the PRISMA extension for scoping reviews (PRISMA-ScR).^16^ The objective, inclusion criteria and method for this scoping review were specified in advance and documented in a protocol.^17^ The electronic search strategy and details for the search process and search terms, including the adjacency (ADJn) operator and truncation used in the search, have been reported in the published protocol.

### Search strategy

This scoping review was conducted between March and July 2021 in accordance with the JBI methodology for scoping reviews which involves a three-step search.^15^ The first search of CINAHL and MEDLINE involved the following keywords: telepresence, (Giraff OR Temi OR VGo OR Double), (robot OR robots OR robotic), (older OR aged OR elderly OR senior). In the second step, we used all keywords and index terms identified from step one to search eight databases: CINAHL, MEDLINE, Cochrane, PsychINFO (EBSCO), Web of Science, ProQuest Dissertations and Theses Global, IEEE Xplore and ACM Digital Library. Google was also searched for gray literature (i.e., organizational reports, newsletters, and other articles not indexed in a library database) using phrases, such as “telepresence robot” OR “robotic telepresence” OR “telepresence technology.” Thirdly, the reference lists of all included articles and reports were screened for additional studies.

The inclusion and exclusion criteria are outlined in Table 1. We included studies that focused on participants aged 65 and older. This review considered studies that provided information about any telepresence robot intervention and outcome on social connection in older adults within care settings. We included studies conducted in formal care settings (staffed by paid employees), such as long-term care (LTC), assisted living, primary care clinics and hospitals, that examined the use of a telepresence robot. All empirical, peer-reviewed publications that examined the use of telepresence robots for older adults were considered. All research designs were considered for this review, including case studies, evaluation studies, empirical studies, quantitative and qualitative designs.

**Table 1.** Inclusion and exclusion criteria.

Inclusion criteria	Exclusion criteria
Articles of user studies with people aged 65 and older	Evidence of user studies with people aged less than 65 years old
Articles focused on telepresence robot(s)	Evidence focused on forms of technology other than telepresence robots
Articles focused on social connection in a healthcare setting with formal care provided by paid staff	Evidence focused on settings without formal care (i.e., home care)
Peer-reviewed journal articles or full reports available on the internet	Only abstracts available
Publications in English	Non-English publications

### Study selection

A bibliographic reference management tool, Mendeley,^18^ was used to ensure that all references and articles were systematically organized. All relevant articles identified relevant were uploaded into Mendeley and duplicates were removed. The review process involved two levels of screening: a title and abstract review followed by a full-text review. In the first level of screening, one research team member screened the titles and abstracts for relevancy. In the second level of screening, the full text of relevant articles was examined for inclusion against the inclusion criteria (See Table 1).

**Figure 2.**
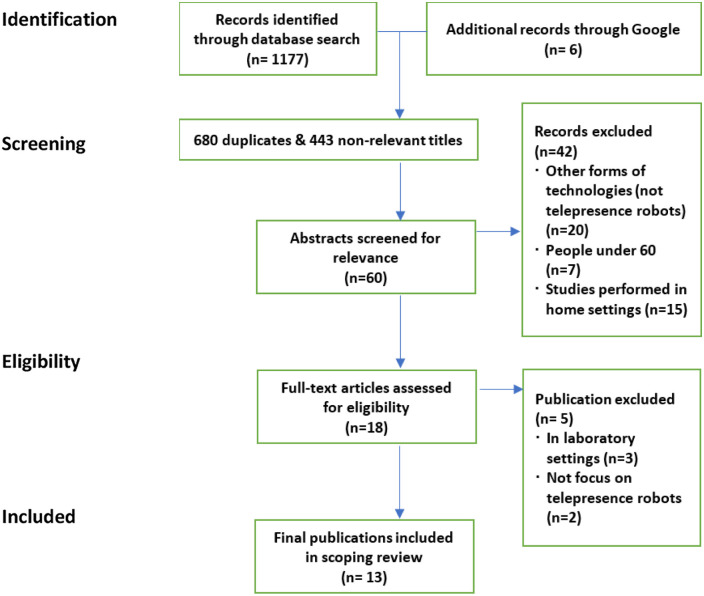
PRISMA flow diagram.

The selection process is presented in the PRISMA flow diagram^15^ (Figure 2). The database search initially yielded 1177 publications and an additional six publications identified through Google search. In the screening process, 680 duplicates and 443 non-relevant titles were removed, which resulted in 60 remained articles. Of these, 42 records were excluded for lack of relevance in terms of the forms of technologies (not telepresence robots) (*n* = 12), participant age (under 65) (*n* = 6), and study settings (home/outside care settings) (*n* = 24). After eligibility assessment on the remaining 18 articles, five studies were excluded. The final review included a total of 13 publications.

### Mapping

Table 2 presents a summary of the selected articles we mapped by 10 domains: author and publication year, country of study, publication type, study type, duration of study, study population, type of healthcare setting, name of telepresence robot, facilitators, and barriers. The 13 publications that met the eligibility criteria for review comprised seven peer-reviewed journal articles, four conference papers, one Master’s thesis, and one book chapter.

**Table 2.** Characteristics of included studies.

Author, year	Country of study	Publication type	Study type	Study duration	Study population	Type of healthcare setting	Telepresence robot name	Facilitators	Barriers
Aaltonen et al., 2017^22^	Finland	Conference paper	Qualitative study	10 weeks	A female resident; her two daughters; nurses	Assisted living	Double	• Sense of presence	• Resident preferred telephone due to hearing issues
								• Remote participation in activities	• Insufficient control for resident
								• Adaptable level of control	• Lack of accessibility
								• Informing other residents and visitors about the video functions of the robot	• Audio problems
								• Scheduling calls	• Privacy and security concerns
								• Clear guidelines	• Incompatible care setting regulations
									• Potential overuse
									• Potential misuse
									• Poor internet connection
Koceski & Koceska, 2016^10^	Macedonia, Europe	Peer-reviewed journal article	Evaluation study	One month	*n* = 35 (30 elderly participants with no severe disability problems (e.g., severe dementia and bedridden); five professional caregivers in the nursing home)	LTC	A developed assistive telepresence robot	• Personalized functions	• Lack of training
								• Easy to use	
								• The possibility of companionship will be enhanced by telepresence robot	
								• Resident’s ability to operate robot	
								• Engagement of multidisciplinary team	
Koceska et al., 2019^28^	Macedonia, Europe	Peer-reviewed journal article	Evaluation study	Unknown	*n* = 31 (26 elderly people; five professional caregivers)	LTC	A developed assistive telepresence robot	• Adaptable level of control	No reported barriers
								• Low-cost design	
								• Sufficient training	
								• Residents not concerned with privacy	
								• Practice sessions	
Korblet, 2019^11^	The Netherlands	Master’s thesis	Qualitative exploratory study	15 minutes demonstration per group; 20–30 minutes interviews per group; total duration about 3 hours	*n* = 11 (three groups of participants over the age of 70 (1) five with dementia (2) three who lived independently but visited the nursing home one to 2 days a week (3) three with physical disabilities)	LTC	Double	• Sense of presence	• Resident preferred telephone due to hearing issues
								• Easy to use	
								• Low-cost design	
								• Sufficient training	• Negative attitude
								• Residents not concerned with privacy	
								• Residents trusted the robot	
								• Positive attitude	• Feelings of doubt towards ability to learn
								• Use robot with trusted individual	
								• Training and knowledge	
Moyle et al., 2013^30^	Brisbane, Australia	Conference paper	Case study design	Unknown	Five research triads: Each included a resident with dementia living in a long-term care home; a family member; and up to two members of the care staff team	LTC	Giraff	• Sufficient training	• Poor internet connection
									• Software and hardware problems
									• Negative attitude
									• Lack of training
Moyle et al., 2014^27^	Queensland, Australia	Peer-reviewed journal article	Mixed-methods study	Four months, with each trial over six to eight weeks	*n*=18 (five residents with mild-to-moderate dementia; six family members; seven staff members)	LTC	Giraff	• Promoted socialization	• Software and hardware problems
								• No need for person with dementia to learn how to use	• Audio problems
								• Adaptable level of control	• Low quality camera
								• Importance of cost-effectiveness analysis	
								• Potential uses other than social connection, for example, to inform family of the resident’s condition by staff	• Expensive
								• Sufficient training	
								• Residents not concerned with appearance	• Privacy and security concerns
								• Positive attitude	
								• Scheduling calls	• Poor internet connection
								• High level of resident engagement during calls	
Moyle et al., 2014^21^	Queensland, Australia	Peer-reviewed journal article	Descriptive qualitative study	Five to seven weeks per trial, number of trials not mentioned	*n*=11 (five residents with mild-to-moderate dementia; six family members)	LTC	Giraff and VGo	• Sense of presence	• Lack of research for residents with moderate and severe dementia in care settings
								• Promoted socialization	• Software and hardware problems
								• Mobility	• Computer incompatibility
								• No need for person with dementia to learn how to use	• Audio problems
								• Easy to use	• Limited mobility
								• Previous experience with similar technology	• Poor internet connection
									• Not viewed as a technology to be used across the trajectory of dementia
									• Not appropriate for residents with cognitive impairment
									• Longer time for resident to feel comfortable with robot
Moyle et al., 2020^26^	Finland	Conference paper	Empirical study	12 weeks	*n*=6 (A 83-year-old female resident with no diagnosed memory illness; two daughters of the resident; three nurses)	Assisted living	Double	• Visualizing caller’s face	• Insufficient control for resident
								• Promoted socialization	• Disruptive to care work
								• Alleviated loneliness	• Audio problems
								• Reduced travel time	• Low quality camera
								• Adaptable level of control	• Privacy and security concerns
								• Easy to use	• Concerns for other residents
								• Positive attitude	• Poor internet connection
								• Ability to increase family member’s role in the care setting	• Lack of training
								• Ethics plan, especially about privacy and control	• Staff felt unskilled
Niemelä et al., 2017^24^	Finland	Peer-reviewed journal article	Empirical study	Three trials, each ranged from five to 12 weeks	In each trial, there were a resident, one or more of her/his family members, the personal nurse of the resident, and other care workers at the ward	Assisted living	Double	• Sense of presence	• Insufficient control for resident
									• Lack of connection indicator
								•Visualizing caller’s face	• Audio problems
								• Remote participation in activities	• Privacy and security concerns
								• Engaged family members in care	• Preferred to keep robot in room
								• Easy to use	• Concerns for other residents
								• Residents not concerned with privacy	• Unclear family and care worker limitations
								• Ability to increase family member’s role in assisted living	• Poor internet connection
								• Scheduling calls	• Limited use
								• Clear guidelines	• Concern about family’s perspective if call is rejected
									• Need for permission from all residents
Niemelä et al., 2019^23^	Finland	A chapter in a book	Empirical study	Three trials, each ranged from five to 12 weeks	Three elderly residents, their family members and three care workers at the first setting and five care workers and a manager at the second setting	Assisted living	Double	• Sense of presence	• Poor ergonomics
								• Mobility	• Privacy and security concerns
								• Adaptable level of control	• Concerns for other residents
								• Personalized functions	• Concern for decreased in person visits
								• Sufficient training	
								• Clear guidelines	
								• Reflecting on future use of robots	
Robinson et al., 2013^29^	Auckland, New Zealand	Peer-reviewed journal article	Cross-sectional study	Over one week	*n* = 26 (10 residents over 71 years old with dementia; 11 family members; five staff members)	LTC	Guide	• Personalized functions	• Too complicated
									• Large size
									• Poor ergonomics
									• Difficult to see screen
									• Not appropriate for residents with cognitive impairment
Reis et al., 2018^31^	Portugal	Conference paper	Proposed roadmap	Unknown	Elderly; family members; care centres' staff	LTC	Proposed to use beam, PadBot2 and Double2	• Suitable setting	• Lack of guidelines
								• Good internet connection	• Lack of planning
								• Sufficient training	
								• Engagement of multidisciplinary team	
								• Establishing an evaluation plan	
Vermeersch et al., 2015^25^	South central Ohio, USA	Peer-reviewed journal article	Descriptive exploratory study	Two weeks	*n* = 14 (13 residents over age 65; one advanced practice registered nurses)	Clinic	RP-7 robot	• Sense of presence	• Software and hardware problems
								• Importance of cost-effectiveness analysis	• Appearance
								• Operations improved with practice	• Expensive

### Theoretical framework

The Consolidated Framework of Implementation Research (CFIR) is a practical tool developed for exploring the implementations of innovations across five domains and 39 constructs.^19^ CFIR is a flexible framework for guiding the analysis for implementation studies. Using CFIR, this scoping review systematically outlines facilitators and barriers to implementing telepresence robots in aged care settings under relevant domains and constructs.

### Synthesis of results

To identifiy themes of relevant barriers and facilitators, we used the CFIR constructs to deductively code the extracted data and inductively analyzed them to allow open codes to emerge. The codes were evaluated, refined and collated into categories to develop themes collectively with our research team. It is multi-disciplinary and multi-sectoral, consisted of 10 members: three people living with dementia, two family partners, one researcher in nursing, one researcher in rehabilitation sciences, and three graduate students. In research meetings, the team took part in analyzing data and sorting according to potential themes. We discussed different interpretations to resolve conflicts. Themes were validated by people living with dementia and family partners. Table 3 summarizes facilitators and barriers to implementing telepresence robots in care settings.

**Table 3.** Summary of the facilitators and barriers mapped onto the constructs within the CFIR domains.

Constructs	Facilitators	Barriers
Domain I: Intervention characteristics		
Evidence strength and quality	No reported facilitators	• Lack of research for residents with moderate and severe dementia in care settings^21^
Relative advantage	• Sense of presence^11,21–25^	• Preference for telephone use due to hearing issues^11,22^
	• Visualizing caller’s face^24,26^	
	• Remote participation in activities^22,24^	
	• Promoted socialization^21,26,27^	
	• Mobility^21,23^	
	• Engaged family members in care^24^	
	• Alleviated loneliness^26^	
	• Reduced travel time^26^	
	• No need for person with dementia to learn how to use^21,27^	
Adaptability	• Adaptable level of control^22,23,26–28^	• Insufficient control for resident^22,24,26^
		• Disruptive to care work^26^
		• Lack of accessibility^22^
	• Personalized functions^10,23,29^	• Lack of connection indicator^24^
Complexity	• Easy to use^10,11,21,24,26^	• Too complicated^29^
Design quality and packaging	No reported facilitators	• Software and hardware problems^21,25,27,30^
		• Computer incompatibility^21^
		• Audio problems^21,22,24,26,27^
		• Low quality camera^26,27^
		• Limited mobility^21^
		• Appearance^25^
		• Large size^29^
		• Poor ergonomics^23,29^
		• Difficult to see screen^29^
Cost	• Low-cost design^11,28^	• Expensive^25,27^
	• Importance of cost-effectiveness analysis^25,27^	
Domain III: Inner setting		
Compatibility	• Potential uses other than social connection, for example, to inform family of the resident’s condition by staff^27^	• Privacy and security concerns^22–24,26,27^
	• Informing other residents and visitors about the video functions of the robot^22^	• Incompatible care setting regulations^22^
		• Preferred to keep robot in room^24^
		• Concerns for other residents^23,24,26^
		• Potential overuse^22^
		• Potential misuse^22^
		• Unclear family and care worker limitations^24^
Available resources	• Suitable setting^31^	• Poor internet connection^21,22,24,26,27,30^
	• Good internet connection^31^	• Lack of training^10,26,30^
	• Sufficient training^11,23,27,28,30,31^	
Domain IV: Characteristics of individuals		
Knowledge and beliefs about the intervention	• Residents not concerned with privacy^11,24,28^	• Negative attitude^11,30^
	• Residents not concerned with appearance^27^	
	• Residents trusted robot^11^	• Limited use^24^
	• Positive attitude^11,26,27^	• Concern about family’s perspective if call is rejected^24^
	• Use robot with trusted individual^11^	• Not viewed as a technology to be used across the trajectory of dementia^21^
	• The possibility of companionship will be enhanced by telepresence robot^10^	• Concern for decreased in person visits^23^
Self-efficacy	• Previous experience with similar technology^21^	• Feelings of doubt towards ability to learn^11^
	• Training and knowledge^11^	• Staff felt unskilled^26^
Individual stage of change	• Operations improved with practice^25^	No reported barriers
Individual identification with organization	• Ability to increase family member’s role in the care setting^24,26^	No reported barriers
Other personal attributes	• Resident’s ability to operate robot^10^	• Not appropriate for residents with cognitive impairment^21,29^
		• Longer time for resident to feel comfortable with robot^21^
Domain V: Process		
Planning	• Scheduling calls^22,24,27^	• Lack of guidelines^31^
	• Clear guidelines^22–24^	• Lack of planning^31^
	• Ethics plan, especially about privacy and control^26^	• Need for permission from all residents^24^
Engaging	• Practice sessions^28^	No reported barriers
	• High level of resident engagement during calls^27^	
	• Engagement of multidisciplinary team^10,31^	
Reflecting and evaluation	• Establishing an evaluation plan^31^	No reported barriers
	• Reflecting on future use of robots^23^	

### Ethical considerations

Research ethics approval and consent to participate was not required for this scoping review because the methodology of the study only consisted of data from articles in public domains. As a team that included academics and clinicians working with people living in care settings, we engaged in team reflection in our regular meetings and used the guidance of the ethical framework “ASK ME” specifically developed for co-research with people with dementia.^20^ The voices of people living with dementia and family partners enriched researchers’ understanding of the topic. The researchers and graduate students also gained skills in the project for engaging people living with dementia and family partners through developing an awareness of the different styles of communication, exploring experiential views, and lived experience perspectives.

## Results

### Characteristics of included studies

**Figure 3.**
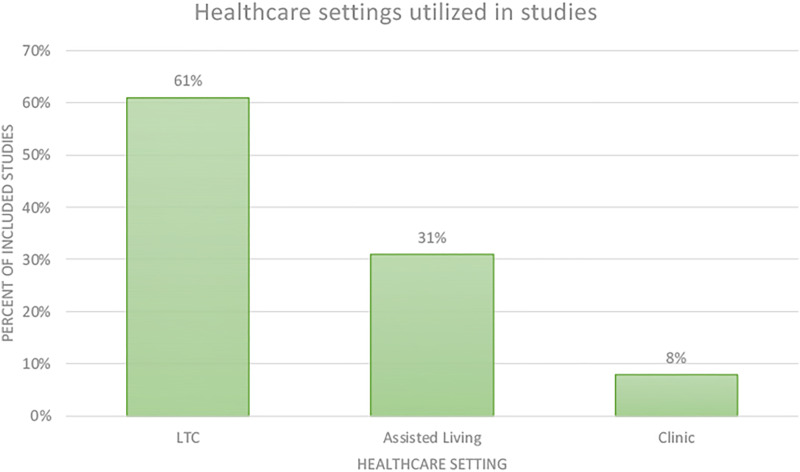
Percentage of healthcare settings utilized in the reviewed studies.

Most studies were conducted in Finland (*n* = 4) and Australia (*n* = 3). Other studies were conducted in the United States (*n*=1), Portugal (*n*=1), New Zealand (*n* = 1), the Netherlands (*n* = 1), and Macedonia (*n* = 2). Many studies were qualitative (*n* = 4), empirical (*n* = 3), or evaluation studies (*n* = 2). Other study types included a case study, a proposed roadmap, a cross-sectional study, and a mixed-methods study. Most studies included residents living in care settings, family members, and care staff. Studies were conducted in long-term care homes (*n* = 8, 61%), assisted living (*n* = 4, 31%), or a clinic (*n* = 1, 8%) (Figure 3). Double was the most commonly used telepresence robot and was used in five studies. Giraff was also a frequently deployed robot (*n* = 3). Guide (*n* = 1), VGo (*n* = 1), and RP-7 (*n* = 1) were less common. An assistive telepresence robot created by a study team was used by the same group in two different studies. A summary of the characteristics of the included studies is provided in Table 2.

### Facilitators and barriers to implementation

After selecting the relevant studies for the review, we identified and mapped the facilitators and barriers in the studies using the CFIR framework. Table 3 summarizes the findings onto 16 constructs within four of the five CFIR domains: (I) Intervention Characteristics, (III) Inner Setting, (IV) Characteristics of Individuals and (V) Process. No constructs were mapped onto (II) Outer Setting. An example facilitator and barrier from the reviewed studies are included for each construct.

The frequency of cited constructs is outlined in Table 4. We categorized the constructs and generated three themes regarding implementation of telepresence robots in aged care settings: relative advantages, perceived risks and problems, and clinical/contextual considerations. The facilitators and barriers mapped onto the constructs are discussed in the relevant themes.

**Table 4.** Frequency of CFIR constructs mapped from the reviewed studies (constructs with no facilitators or barriers were omitted).

CFIR domains and constructs	Facilitators n (%) of studies	Barriers n (%) of studies
I. Intervention characteristics		
Evidence strength and quality	None	1 (7.7%)
Relative advantage	8 (61.5%)	2 (15.4%)
Adaptability	7 (53.8%)	4 (30.8%)
Complexity	4 (30.8%)	1 (7.7%)
Design quality and packaging	None	8 (61.5%)
Cost	4 (30.8%)	2 (15.4%)
II. Outer setting	None	None
III. Inner setting		
Compatibility	3 (23.1%)	5 (38.5%)
Available resources	5 (38.5%)	5 (38.5%)
IV. Characteristics of individuals		
Knowledge and beliefs about the intervention	6 (46.1%)	5 (38.5%)
Self-efficacy	2 (15.4%)	2 (15.4%)
Individual stage of change	1 (7.7%)	None
Individual identification with organization	2 (15.4%)	None
Other personal attributes	1 (7.7%)	2 (15.4%)
V. Process		
Planning	5 (38.5%)	2 (15.4%)
Engaging	4 (30.8%)	None
Reflecting and evaluation	2 (15.4%)	None

After mapping the constructs, we classified our findings using three themes: (1) relative advantages, (2) perceived risks and problems and (3) clinical/contextual considerations.

### Theme 1: Relative advantages

This theme encompasses the reported advantages telepresence robots have over other means of communication. The CFIR construct relative advantage (intervention characteristics) falls within this theme.

*Domain I. Intervention characteristics: relative advantage.* Implementation was facilitated as telepresence robots provided pleasure and a sense of connection when compared to other technology (e.g., telephone) due to the live video connection. Reported benefits of the video function include viewing the person on the call^11,21,24,26^ and the surrounding environment,^21^ which generated feelings of presence of the other person,^21–25^ promoted socialization,^26^ and encouraged longer conversations.^21^ One participant reported they preferred the robot over other technologies because “in this case you actually see the other person. It’s completely different.”^11(p: 20)^ However, some residents preferred a regular phone conversation^22^ and felt the robot was unnecessary due to other available technologies.^11^ One participant reported “I don’t think it’s necessary, because nowadays there are so many robots and technical machines… For me it’s not necessary.”^11(p: 20)^

The robot allowed viewing and remote participation of activities that would not be as well facilitated by a regular phone call^22,24^ and reduced travel time required for in-person visits.^26^ The benefits of the telepresence robot over other video platforms such as Skype were the ability to move the robot^21^ and the ease of use of the robot, which was particularly important for residents with dementia.^21,27^ Furthermore, the robot allowed family members to engage more effectively in the care of the resident by improving the understanding of the health condition of the resident, assisting in the planning of care, and helping communicate issues to care workers, thereby improving care.^24^

### Theme 2: Perceived risks and problems

This theme encompasses perceived risks (cost, privacy, security, overuse or misuse) and issues (poor internet connection, lack of skill among users, and other technological difficulties) related to the utilization of telepresence robots in care settings. The CFIR constructs of design quality and packaging (intervention characteristics), cost (intervention characteristics), compatibility (inner setting), and available resources (inner setting) fall within this theme.

*Domain I. Intervention characteristics: design quality and packaging.* Barriers related to the robot’s design quality include technical difficulties with the software and hardware.^21,25,27^ Computer incompatibility and low camera quality were also reported challenges.^21,26,27^ Issues with audibility were frequently reported including difficulties setting audio volume^22,24,26,27^ and poor audibility compared to cell phones.^24,26^ As a result, residents in one study “preferred their mobile phones for quick checks and also for longer discussions because of better audibility.”^24(P: 5)^ Some users felt the robot was unnecessarily large for its function. One participant noted the robot seems “big for the capabilities … you could have an I-pad or a big touch screen that would do the same things as this does. Really all this is just supporting it…it’s not actually doing anything is it?”^29(p: 38)^ Other users felt the robot had a cold and mechanical appearance,^25^ and lacked proper ergonomics.^23,29^

*Domain I. Intervention characteristics: cost.* Considerations of performing a cost-effectiveness analysis were reported as an important feature to justify the use of more expensive telepresence robots.^25,27^ However, low-cost robots have been developed and validated.^11,28^

*Domain III. Inner setting: compatibility.* More barriers were identified in this construct than facilitators. Barriers related to compatibility highlight perceived risks to privacy and security. Navigating the robot through the care setting poses the risk of a family member on the call witnessing and potentially recording private situations or conversations involving the resident or others in the care setting.^22–24,26^ Family members and care workers were also concerned about the resident’s privacy,^26^ including concerns of invading the personal space of the resident.^24^ Other barriers include concern for other residents’ negative responses to the wandering robot^23,24,26^ and institutional regulations that prohibit mobility of the robot in common areas.^22^ However, some settings permitted the robot’s navigation in common areas and took the steps to inform others that the robot could be transmitting video.^22^ Another facilitator highlighted the potential use of the robot for other tasks in addition to social connection.^27^ Additional barriers include potential overuse or misuse of the robot’s call function,^22^ preference to keep the robot in the room^24^ and unclear limitations of the role of family and staff members in calls.^24^

*Domain III. Inner setting: available resources.* Five studies reported that sufficient training for staff, residents, and family members facilitates the implementation of telepresence robots.^11,23,27,28,31^ Training should “differ according to the profile of the participants, adapting to their capacities and limitations as well as to their ability to use technological equipment.”^31(p: 3)^ Another facilitator was the availability of a suitable setting with good internet connection.^31^ Poor internet connection was reported as a barrier in five studies.^21,22,24,26,27^ Barriers relating to a lack of training were reported in two studies.^10,26^

### Theme 3: Clinical/contextual considerations

This theme encompasses the importance of adequate planning and creation of guidelines while considering the user population and their specific needs for successful implementation. The following CFIR constructs fall under this theme: evidence strength and quality (intervention characteristics), adaptability (intervention characteristics), complexity (intervention characteristics), knowledge and beliefs about the intervention (characteristics of individuals), self-efficacy (characteristics of individuals), individual stage of change (characteristics of individuals), individual identification with organization (characteristics of individuals), other personal attributes (characteristics of individuals), planning (process), engaging (process) and reflecting, and evaluation (process).

*Domain I. Intervention characteristics: evidence strength and quality.* One study suggested further research investigating the benefits of the telepresence robot for residents with dementia over time is needed. This suggestion is based on the advantages of the robot being mediated by the resident’s level of cognitive function.^21^

*Domain I. Intervention characteristics: adaptability.* Primary facilitators identified in previous studies were the different levels of settings over the robot’s control^28^ and the ability to use the robot’s mobility as suited to the user’s needs.^23,26,27^ Furthermore, the functions of the robot could be personalized to the resident’s needs^10,29^ and allow remote engagement in social events within the care setting.^23^ However, family members and care workers felt that residents should have more control over the robot, including having the ability to accept or reject calls^24,26^ and improving privacy by having the option to prevent calls coming through.^22,24^

Although allowing residents to have increased control was considered a facilitator in some studies, it may raise some concerns on the ability of certain residents (e.g., those with cognitive impairments) to operate the robot. Robot use should be adapted in a way to maximize control while considering the technological abilities of the resident. For example, for participants with memory problems, it was suggested “turning off the robot might not be feasible but using an easy-to-use ‘30-min privacy’ button could be practical.”^22(p: 56)^ Adaptations suggested were to improve the robot by including an indication of when a connection is open,^22,24^ making the caller’s identity visible before starting the call,^22^ and linking the robot to family members’ smart phones to allow a call at the request of the resident.^22^

*Domain I. Intervention characteristics: complexity.* Most users reported the telepresence robot was easy to operate,^10,11,21,24,26^ although the Guide robot was critiqued for being too complicated in one study.^29^

*Domain IV. Characteristics of individuals: knowledge and beliefs about the intervention.* Residents reported not being concerned with privacy issues^11,24,28^ or the appearance of the robot.^27^ Residents did not have trust issues surrounding the robot^11^ and reported positive attitudes toward the technology.^10,11^ Most participants in one study “expected that their families would enjoy interacting with the robot and that they would find the robot ‘interesting’.”^11(p: 22)^ Residents may be more likely to accept and utilize the robot if it is used with someone they trust, such as a family member.^11^

Family members and care workers had positive attitudes toward the robot and its impact on residents.^26,27^ However, some residents had a negative attitude toward the robot in one study^11^ and felt the robot should only be used to connect with family members.^24^ Care workers were concerned about the family’s response if calls were ended or rejected.^24^ Other barriers include limited use with residnets with dementia,^21^ concern with a resulting decrease in physical visits by family members,^23^ and privacy in some situations.^22^

*Domain IV. Characteristics of individuals: self-efficacy.* Facilitators included previous experience with video platforms^21^ and knowledge on how to operate the robot.^11^ For example, one family member reported “I’m used to talking on Skype. I’m used to remote connection for meetings and all sorts of things. So, no, I wasn’t concerned. I had a general idea of how it would work.”^21(p: 4)^ However, residents with dementia felt that they were unable to learn or understand the robot^11^ and some care workers felt unskilled.^26^

*Domain IV. Characteristics of individuals: individual stage of change.* Only one facilitator was reported: skill using the robot improved with practice.^25^

*Domain IV. Characteristics of individuals: individual identification with organization.* A facilitator discussed in two studies was the ability of the robot to increase family participation and care planning in the care setting, resulting in the family feeling more connected to the care setting.^24,26^

*Domain IV. Characteristics of individuals: other personal attributes.* Two studies reported the robot may not be appropriate for residents with cognitive impairments.^21,29^ One care worker expressed concern about residents with memory problems using the robot, and another staff member felt that other residents without memory problems within the care setting might be better suited to interact with this technology.^29^ Furthermore, residents may require longer periods of time to feel comfortable with the robot due to lack of previous experience with similar technology,^21^ while some residents had the ability to operate the robot themselves.^10^

*Domain V. Process: planning.* Three studies reported that pre-scheduling calls to maintain privacy and helping residents prepare for a call facilitated the implementation of telepresence robots in care settings.^22,24,27^ Other facilitators include developing clear written rules surrounding calls,^22^ considering and addressing ethical issues^23,26^ and meeting the unique needs of particular residents.^24^ Barriers included a lack of defined rules,^31^ and the need to obtain the signed permission of all residents to use the robot in common spaces and under “surveillance mode.”^24^ Scheduling the date for the robot installation to avoid disturbing daily routines of the care setting was also suggested.^31^

*Domain V. Process: engaging.* A high level of engagement of the residents during calls was reported^27^ and free practice sessions were considered beneficial.^28^ Care workers could also engage and assist with the robot when needed.^10^

*Domain V. Process: reflecting and evaluation.* Establishing an evaluation plan was recommended.^31^ Reflecting on the future use of robots was also reported after utilizing the robots in the care setting.^23^

## Discussion

This study identified various facilitators and barriers to implementing telepresence robots in aged care settings. Three key themes identified were relative advantages, perceived risks and problems, and clinical/contextual considerations

This review showed residents, family members, and care workers preferred telepresence robots over other communication means. The implementation will be more likely to succeed if users recognize a definite advantage in the effectiveness of the intervention.^32^ It is critical for every stakeholder to have clear ideas about the benefits of telepresence robots, such as providing the feeling of family members’ presence,^21–25^ reducing loneliness of residents^10^ and being a less skill-demanding device for older adults.^21,27^ For instance, implementors can adopt different strategies to demonstrate the benefits to different parties in the planning phase, such as showing videos about residents' reactions to the robots to family members, care workers, and other aged care settings. For older adults who are not familiar with technology, customized strategies are essential to increase their motivation to use new technology. Step-by-step strategies from exploring perceptions, explaining concepts, responding to concerns, to showing the relevant relative advantages of telepresence robots to stakeholders may facilitate the acceptance and adoption of the robots.^33^ In addition, no studies has explored the perspectives of leaders and administrative mangers, who are key stakeholders in implementation. Effective implementation requires acknowledgment from all relevant stakeholders. For future implementation, planners should include and motivate leaders of the aged care settings to learn about the advantages of telepresence robots.

This review revealed consistent concerns of telepresence robots over privacy. For example, the possibilities of witnessing residents’ personal and private situations, overhearing workers’ conversations, and recording videos by remote users.^23,24^ Lack of security and resultant discomfort may create active and passive resistance toward implementation and are inhibitors of technology readiness. Technology readiness can be defined as “people’s propensity to embrace and use new technologies for accomplishing goals in home life and at work.”^34(p: 308)^ If inhibitors exist, individuals anticipate risks rather than benefits regarding a new technology. This situation is negatively related to their intention to use the technology.^35^ To overcome the situation, assurance and firm support are critical enablers to implementing telepresence robots. Moreover, having adequate communication and various platforms for stakeholders to voice their concerns is helpful during the planning phase. Engaging key stakeholders to co-create usage guidelines and restrictions for the implementation of telepresence robots may also enhance empowerment and alleviate perceived risks and concerns.

The theme of perceived risks and problems also encompasses the experiences of technical issues during the implementation of telepresence robots in aged care settings, for example, internet connections,^24,26,27,30^ audio volumes,^22,24,26,27^ screen tilting,^29^ and software issues.^27^ In all studies that encountered these problems, research teams provided immediate technical support. Timely support is a critical factor in facilitating the implementation of innovation.

Missing from the studies was any mention of the transitional phase when the research team has withdrawn and technical support has been removed. Rather they only reported findings discovered during the relatively short study time period. Implementors can investigate how to ensure a gradual reduced need for technical support from the research team during the transition period. Otherwise, unresolved technical issues may impede stakeholders to continue using the robots. Successful implementation includes a sustainble plan for the adoption of telepresence robots in the aged care settings.

Furthermore, implementors should optimize the facilitation provided to residents during the use of robots. Empowering and building resident self-efficacy in their ability to utilize the implementation of teleprescence robots heightens their confidence, which will in turn increase the probability that the intervention will be accepted.^36^ Previous research highlighted that understanding how the robots work and how to operate them plays a critical role for residents to accept and use them.^11^ The balance between facilitating the use of telepresence robots and respect for privacy is an ongoing challenge for implementation.

Our results also highlighted the adaptability of telepresence robots in aged care settings. To facilitate the implementation in settings that serve mostly older populations, the adaptation of robots would be different from business and educational settings. There would be special considerations on robots for older populations in the articles included which can be possible facilitators to implementation, for example, adjustable heights to facilitate use by residents who are in wheelchairs, appropriate audio volume for older adults with hearing difficulties, optimal screen size for those with impaired eyesight, and additional reminder functions. The availability of these adaptive components can facilitate the implementation of telepresence robots in aged care settings.

Finally, this review highlighted the logistical challenges and facilitators in implementing telepresence robots in aged care settings for clinical/contextual considerations. Having practical and well-planned training is an essential enabler for successful implementation.^37^ With diverse organizational cultures and structures in aged care settings, most articles reviewed provided training specific to different subgroups in their studies, for example, family members and older adults.^11,27,28^ However, the articles did not mention the degree of involvement of key stakeholders in designing training. Engaging all stakeholders early in the implementation process enhances success^38^ and can also foster cultural understanding to tailor appropriate training. In addition, there were doubts on whether using telepresence robots would interrupt existing workflow in two articles.^22,24^ Tackling this issue early by involving stakeholders in the planning phase can avoid creating a barrier to implementation and turn the issue into an opportunity to facilitate a better implementation process. Implementors can leverage recommendations from existing articles to respond to the concern of interrupting existing workflows, such as pre-scheduling the meeting time of residents with their family members^22,24,27^ and outlining clear guidelines and boundaries for robotic uses by staff and family members.^22,24,31^

The findings in this review are comparable to the findings reported in the 2017 review of telepresence robots^14^ and suggest that telepresence robots may facilitate social connection and benefit all older adults in care settings, and not just older adults with dementia. Furthermore, the findings are also comparable to studies investigating other types of robots in care settings. For example, a 2019 scoping review investigated the benefits and barriers of utilizing social robot PARO in care settings for older people with dementia and reported three key benefits and three key barriers.^39^ Similar to telepresence robots, social robots facilitated social connection and promoted positive mood. Although our review did not identify a specific reduction in negative emotion and behavioral symptoms as reported in the PARO review, these outcomes may be possible as a result of telepresence robots’ ability to facilitate social engagement and should be further investigated. There is substantial overlap in the reported barriers in the PARO review as well, specifically cost, stigma, and ethical issues. Further similarities can be identified in a recent scoping review that used the CFIR framework to map barriers and facilitators for the use of social robots for older adults and people with dementia.^40^ Similar facilitators include sense of presence, ease of use, mobility, and practice with the robot. Similar barriers include audio issues, connection problems, hardware problems, and negative attitude. The overlap across different types of robots provides additional motive for researchers and care staff to further investigate robotic technology because beneficial findings and recommendations may be applicable to a range of robots. Researchers can learn from each other to optimize methodology and establish guidelines to use robotic technology to assist and support older adults in care settings.

### Future research and practical implications

First, we recommend using a comprehensive implementation framework such as CFIR to plan a structured approach, considering a broad range of strategies and anticipated barriers. None of the reviewed studies used an implementation framework to ensure a systematic approach for evaluating the process nor did they consider all factors for implementing innovations. The use of a framework to plan, document and evaluate the barriers and challenges may prevent repetition of these problems in subsequent research. Documenting facilitators and successes helps future implementors make informed decisions to improve the implementation process. Therefore, future research should consider applying a theoretical and systematic framework, such as CFIR, to guide, document, and evaluate the research process from the planning phase.

Second, future research should investigate what helps facilitate the implementation process that meets the specific needs of residents living with early to late stages of dementia. In our review, researchers did not explore differences in implementing telepresence robots for residents in different stages of dementia, for example, their acceptance and responses to the robots.

Third, safety is a crucial area of consideration during implementation. However, the articles that we reviewed did not put adequate emphasis on this area. In addition the issue of preventing physical collisions of robots with objects in the environment, future research needs to explore other safety issues (e.g., robot falling and breaking screen) and identify practical ways (e.g., protective screen) to ensure the safety of both users and telepresence robots. Safety guidelines should be written in appropriate languages for multicultural workplaces; user-friendly instruction and reminder cards should be provided to users for quick references.

In addition, future research can investigate the significance of support and acceptance of senior leaders and managers on the implementation of telepresence robots. Findings can provide insights from an organizational level. The findings can also allow implementors to identify implementation strategies that balance the needs of different stakeholders in an organization, resulting in a smoother implementation process.

Finally, the studies included here were mainly short-term studies. As adopting an innovation requires time, especially for older adults, future research might involve conducting a longitudinal study as was suggested by previous research.^12^ This would allow a comprehensive observation of different stakeholders and participants over an extended period. With longer implementation and trial periods, assumptions by staff, family members, and residents may be reduced. A longer implementation period would allow for more education and engagement time of staff and leadership. Buy-in and engagement among staff and leadership may increase with a better understanding of the robot by creating opportunities for learning and sharing of information. Moreover, technical malfunctions may not be addressed in short-term studies, which may negatively affect the process of implementation and technology adoption. Future studies focused on the long-term will better identify the feasibility of implementation of the robots in everyday practice, while also exploring the comfort levels among residents with various stages of dementia as they interact with telepresence robots.

### Strengths and limitations

The research was strengthened by a transdisciplinary approach, which facilitated the exchange and integration of knowledge and perspectives particularly between academic and non-academic team members (i.e., people living with dementia and family partners).^41–43^ Collaborating with non-academic members was particularly helpful for the academic members when interpreting the findings of reviewed studies from user perspectives, understanding the benefits and challenges of adopting telepresence robots in aged care settings, and generating implications for future research and implementation of telepresence robots in aged care settings. People living with dementia and family partners provided helpful insights and enriched data analysis.

This review contributed to knowledge related to the implementation of telepresence robots for older adults in care settings. A scientific and valuable implementation tool, CFIR, was used to guide the review of studies and facilitate a systematic and clear presentation of results. However, this review has several limitations. We acknowledged that there were other telepresence robots we were unaware of (e.g., Beam,^44^ Cutii,^45^ and Kompai^46^) when identifying search terms. Moreover, we did not include general terminology used to refer to telepresence robots in our search terms, for example, “tele-operated” or “social robot.” Missing these keywords for our search might have limited our search results. In addition, we only included English-language publications and most of the studies were short-term studies with limited sample sizes. For the use of the implementation tool, some domains of CFIR did not apply to the studies.

## Conclusion

Drawing from the CFIR framework, we systematically identified and presented the facilitators and barriers to implementing telepresence robots. The key facilitators to telepresence robot adoption are: a feeling of physical presence, ease of use, mobility, and training. The barriers to implementation are: cost, privacy issues, internet connectivity, and workflow. Future research should investigate the role of leadership support in implementation and practical strategies to overcome barriers to technology adoption in aged care settings. Further research is needed on under-examined aspects of implementation using a systematic implementation framework.
